# Angiopoietin‐Like 2 Promotes Atherogenesis in Mice

**DOI:** 10.1161/JAHA.113.000201

**Published:** 2013-06-21

**Authors:** Nada Farhat, Nathalie Thorin‐Trescases, Maya Mamarbachi, Louis Villeneuve, Carol Yu, Cécile Martel, Natacha Duquette, Mathieu Gayda, Anil Nigam, Martin Juneau, Bruce G. Allen, Eric Thorin

**Affiliations:** 1 Department of Pharmacology, Université de Montréal, Montreal, Quebec, Canada (N.F., C.Y., C.M., E.T.); 2 Montreal Heart Institute, Centre de recherche, Montreal, Quebec, Canada (N.F., N.T.T., M.M., L.V., C.Y., C.M., N.D., M.G., A.N., M.J., B.G.A., E.T.); 3 Department of Medicine, Université de Montréal, Montreal, Quebec, Canada (M.G., A.N., M.J., B.G.A.); 4 Department of Surgery, Université de Montréal, Montreal, Quebec, Canada (E.T.)

**Keywords:** adhesion molecules, aging, CAD, freshly isolated mouse endothelial cells, inflammation, mouse model of atherosclerosis

## Abstract

**Background:**

Angiopoietin like‐2 (angptl2), a proinflammatory protein, is overexpressed in endothelial cells (ECs) from patients with coronary artery disease (CAD). Whether angptl2 contributes to atherogenesis is unknown. We tested the hypothesis that angptl2 promotes inflammation and leukocyte adhesion onto ECs, thereby accelerating atherogenesis in preatherosclerotic dyslipidemic mice.

**Methods and Results:**

In ECs freshly isolated from the aorta, basal expression of TNF‐α and IL‐6 mRNA was higher in 3‐month‐old severely dyslipidemic mice (LDLr^−/−^; hApoB_100_^+/+^ [ATX]) than in control healthy wild‐type (WT) mice (*P*<0.05) and was increased in both groups by exogenous angptl2 (100 nmol/L). Angptl2 stimulated the adhesion of leukocytes *ex vivo* on the native aortic endothelium of ATX, but not WT mice, in association with higher expression of ICAM‐1 and P‐selectin in ECs (*P*<0.05). Antibodies against these endothelial adhesion molecules prevented leukocyte adhesion. Intravenous administration of angptl2 for 1 month in preatherosclerotic 3‐month‐old ATX mice increased (*P*<0.05) total cholesterol and LDL‐cholesterol levels, strongly induced (*P*<0.05) the expression of endothelial proinflammatory cytokines and adhesion molecules while accelerating atherosclerotic lesion formation by 10‐fold (*P*<0.05). Plasma and aortic tissue levels of angptl2 increased (*P*<0.05) with age and were higher in 6‐ and 12‐month‐old ATX mice than in age‐matched WT mice. Angptl2 accumulated to high levels in the atherosclerotic lesions (*P*<0.05). Finally, angptl2 was greatly expressed (*P*<0.05) in ECs cultured from CAD patients, and circulating angptl2 levels were 6‐fold higher in CAD patients compared with age‐matched healthy volunteers.

**Conclusions:**

Angptl2 contributes to the pathogenesis of atherosclerosis.

## Introduction

Angiopoietin‐like (angptl) proteins are a new family of angiogenic factors composed of 8 members (angptl1 to 8).^1–3^ Of these, angptl3,^4–5^ angptl4,^6^ and angptl6^7–8^ regulate lipid and energy metabolism and could therefore contribute to the regulation of cardiovascular functions as well as influence the progression of cardiovascular diseases, including atherosclerosis.^8–9^ Angptl2 is a circulating glycoprotein with abundant expression in the heart, adipose tissue, lung, kidney, and skeletal muscle.^10–11^ Its expression is stimulated by hypoxia,^8,11^ and it induces angiogenesis and endothelial cell (EC) migration.^2,11–12^ Overexpression of angptl2 is proinflammatory in keratinocytes, in adipose tissue, and in ECs.^1,11^ In both mice and humans, circulating levels of angptl2 correlate with inflammation, adiposity, and insulin resistance.^11,13^ We previously reported a 4‐fold increase in mRNA levels of angptl2 in ECs isolated from arteries of active smokers with severe coronary artery disease (CAD) compared with nonsmokers,^14^ whereas angptl2 has recently been associated with synovial inflammation in rheumatoid arthritis,^15^ chronic inflammation in dermatomyositis,^16^ cancer,^17–18^ and abdominal aortic aneurysms.^19^ In addition, plasma levels of angptl2 were higher in Japanese patients with CAD than in healthy subjects and correlated with the severity of the disease.^8,11^ Collectively, these data suggest that angptl2 promotes inflammation, but its contribution to the development of chronic endothelial/vascular inflammation leading to atherosclerosis remains to be demonstrated.

The present study was designed to determine the role of angptl2 and by which molecular mechanism it could participate in the pathogenesis of the atherosclerotic process in severely dyslipidemic LDLr^−/−^; hApoB^+/+^ (ATX) mice. We report that angptl2 induces a proinflammatory response in freshly isolated ECs and triggers leukocyte adhesion onto the native endothelium of preatherosclerotic young ATX mice, the latter being a necessary primary step of atherogenesis,^20^ but not in healthy young mice. We demonstrate that angptl2 is abundant in the atherosclerotic plaque that spontaneously develops with age in ATX mice, that ECs, but not vascular smooth muscle cells (VSMCs), produce angptl2, and that angptl2 binds predominantly to VSMCs. Importantly, chronic administration of angptl2 in preatherosclerotic young ATX mice increases total cholesterol and LDL‐cholesterol levels, strongly induces the expression of endothelial proinflammatory cytokines and adhesion molecules, and accelerates the formation of atherosclerotic lesions. Finally, circulating angptl2 levels are much higher in CAD patients compared with healthy subjects. Altogether, our data are the first to suggest that angptl2 causally contributes to the pathogenesis of atherosclerosis.

## Methods

### Animal Models

Wild‐type (WT) C57Bl/6 and knockout/transgenic severely dyslipidemic LDLr^−/−^; hApoB^+/+^ (ATX) male mice were fed a normal diet.^21–22^ Experiments were performed in agreement with the guidelines for the Care and Use of Laboratory Animals of Canada and following approbation by the animal care committee of the Montreal Heart Institute. Mice were anaesthetized with 44 mg/kg ketamine and 2.2 mg/kg xylazine and ventilated.

In 3‐month‐old WT and ATX mice, the aortas and spleens were used to assess the adhesion of leukocytes onto the native endothelium,^22^ mRNA expression of adhesion molecules and inflammatory genes, and protein expression of adhesion molecules in freshly isolated ECs and leukocytes.

### RNA Extraction and Real‐Time Quantitative PCR

Total RNA was extracted from leukocytes and from freshly isolated ECs, scraped with a blade from longitudinally opened segments of the thoracic aorta, using an RNeasy Mini Kit (Qiagen, Toronto, Ontario, Canada). Total RNA was reverse‐transcribed, and the qPCR reactions were performed using a MxPro3000 platform (Agilent, Mississauga, Ontario, Canada). The primers of target genes (angptl2, TNF‐α, IL‐6, ICAM‐1, P‐Selectin, CD18, CD62L, CD162) were designed using Clone Manager software (Table S1).

### Leukocyte Adhesion to Native Endothelium

Fresh aorta sections with the endothelium facing up were incubated for 30 minutes with or without 100 nmol/L of recombinant angptl2 (see online supplement), 10 U/mL of thrombin (Sigma‐Aldrich, Oakville, Ontario, Canada), anti‐P‐selectin (1:50, #sc6943; Santa Cruz Biotech, Santa Cruz, CA), anti‐ICAM‐1 (1:50, #sc1511; Santa Cruz Biotech, Santa Cruz, CA), goat‐IgG (1:50, #sc2028; Santa Cruz Biotech, Santa Cruz, CA,) or rat‐IgG (1:50, #sc2026; Santa Cruz Biotech,). Leukocytes were radiolabeled and exposed to the endothelial surface of the aorta sections as described previously.^22^ The number of adherent leukocytes to the endothelium was expressed per surface area of the segment (adherent cells/mm^2^ of endothelium surface area).

### Expression of Adhesion Molecules on Leukocytes

Protein expression of CD18 (#553293), CD62L (#553151), and CD162 (#555306), all from BD Biosciences, Mississauga, Ontario, Canada, was analyzed by imaging flow cytometry (AMNIS, Markham, Ontario, Canada). All data were collected with INSPIRE software and analyzed with IDEAS analytical software.

### Chronic Infusion of Angptl2

Preatheroclerotic dyslipidemic 3‐month‐old ATX mice were infused (intravenously) with purified recombinant angptl2 (0.944 μg/μL) or TBSE (50 mmol/L Tris‐base, 150 mmol/L NaCl, 1 mmol/L EDTA) using osmotic minipumps (0.11 μL/h, volume 100 μL; Alzet, Cupertino, CA), for 1 month. We targeted a concentration of angptl2 at equilibrium of ≈150 ng/mL, equivalent to twice the plasma concentration measured in 12‐month‐old atherosclerotic ATX mice (estimated by ELISA [antibodies‐online.com], data not shown). Mice were euthanized at 4 months of age after 1 month of infusion; the aortas were dissected to measure atherosclerotic lesions (in unstained aortas, using GIMP 2.6, http://www.gimp.org), ECs were scraped from the aortas to extract mRNA, and plasma samples were collected to quantify total cholesterol, HDL‐cholesterol, LDL‐cholesterol, and triglycerides levels (Clinical Biochemistry Laboratory, Montreal Heart Institute).

### Plasma, Aortic, and Plaque Angplt2 Protein Levels

Plasma, aortic, and plaque Angplt2 protein levels were measured in 3‐, 6‐, 9‐, and 12‐month‐old WT and ATX mice by Western blot using a goat anti‐angptl2 antibody (dilution 1:200, #AF2084; R&D, Minneapolis, MN).

### Immunofluorescence

Fixed tissues (longitudinally open aortic segments or frozen sections) were incubated with goat anti‐angptl2 (1:50, R&D) or rat anti‐CD31 (1:20, #ab7388‐50; Abcam, Cambridge, MA) and with the appropriate secondary antibody (Alexa fluor‐555 donkey anti‐goat #A21433, Molecular Probes, Burlington, Ontario, Canada; or Alexa fluor‐647 anti‐rat #712‐495‐153, Jackson Immunoresearch, West Grove, PA). Fluorescence was visualized by confocal microscopy (Zeiss, Toronto, Ontario, Canada).

### Cell Culture

Experiments were performed in (1) human internal mammary artery endothelial cells (hIMAECs), (2) human umbilical vein endothelial cells (HUVECs; Lonza, Mississauga, Ontario, Canada), and (3) VSMCs (Lonza). hIMAECs were isolated from discarded segments of IMA from patients undergoing coronary artery bypass graft and were cultured as previously described.^23^

### Western Blots

To measure the secretion of endogenous angptl2 into the culture medium, hIMAECs, HUVECs, or VSMCs were cultured in serum‐free medium for 16 hours. The proteins (50 μg of protein from the cell lysate, 20 μL from the concentrated culture medium, or 2 μL from the purified recombinant protein) were resolved by electrophoresis, transferred to nitrocellulose, and revealed using a goat anti‐angptl2 antibody (dilution 1:200; R&D).

### Fluorescent Immunocytochemistry

hIMAECs, HUVECs, and VSMCs were cultured for 24 hours on coverslips, washed, fixed, and incubated with an angptl2‐specific antibody (1:50; R&D) and then with an Alexa fluor‐488 donkey anti‐goat secondary antibody. Fluorescence was visualized using a confocal microscope.

### Angptl2‐Gaussia Luciferase‐GST Construct

To visualize the binding of angptl2 on the cell surface, cultured ECs and VSMCs were stimulated with angptl2‐luciferase protein secreted by HEK‐293 cells transfected with angptl2‐luciferase‐pcDNA3.1 or with luciferase‐pcDNA 3.1 (as a negative control). For live cell imaging, VSMCs or ECs (HUVECs and hIMAECs) were stimulated 10 minutes with HEK‐293‐angptl2‐luciferase‐ (100 nmol/L) or HEK‐293‐luciferase‐transfected medium. The fraction of angptl2‐luc binding to the cell surface was monitored by the activity of luciferase, using a Gaussia Luciferase assay kit (New England Biolabs, Whitby, Ontario, Canada) with a confocal microscope (Zeiss) before and immediately after the addition of the luciferase substrate. A 450‐nm laser was used, and images (512×512), acquired at 0.5‐second intervals, were collected with a 63x/1.4 plan apochromat Oil DIC objective.

### Plasma Angptl2 Concentration in Human

A sample of blood was drawn from CAD patients (n=11) and healthy volunteers (n=6) (characteristics of subjects in the Table), all members of the Prevention center of the Montreal Heart Institute. This study was approved by the Montreal Heart Institute ethics committee (Project#11‐1328). All donors signed an informed consent form. Circulating human angptl2 levels were measured using a commercial ELISA kit (#ABIN41096; antibodies‐online.com). CAD patients were treated as follows: 91% (9 of 11) with statins, 55% (6 of 11) with antiplatelet agents, 45% (5 of 11) with β‐blockers, 36% (4 of 11) with angiotensin receptor antagonists, 27% (3 of 11) with ACE inhibitors, and 9% (1 of 11) with diuretics. Age‐matched healthy volunteers received no medication.

**Table 1.** Clinical Profiles of Male Subjects in Whom Plasma Angptl2 Concentration Was Measured

	Healthy Volunteers (n=6)	CAD (n=11)
Age, y	60±3	60±4
Glucose, mmol/L	5.2±0.1	5.6±0.2
Triglycerides, mmol/L	1.4±0.2	1.1±0.2
Total cholesterol, mmol/L	4.8±0.2	3.8±0.2*
HDL‐cholesterol, mmol/L	1.5±0.1	1.3±0.1
LDL‐cholesterol, mmol/L	2.9±0.2	2.0±0.2*
BMI, kg/m^2^	23.9±0.9	28.2±1.1*
SAP, mm Hg	118±5	132±5
DAP, mm Hg	72±3	80±2*

Data are mean±SEM of n=6 healthy individuals and n=11 CAD patients. Angptl2 indicates angiopoietin like‐2; CAD, coronary artery disease; HDL, high‐density lipoprotein; LDL, low‐density lipoprotein; BMI, body mass index; SAP, systolic arterial pressure; DAP, diastolic arterial pressure.

* *P*<0.05 vs healthy volunteers.

Experimental details concerning the cloning, expression, and purification of recombinant angptl2‐GST (Figure S1) plus all protocols used in this study are provided in the online supplement.

### Statistical Analysis

**Figure 1.**
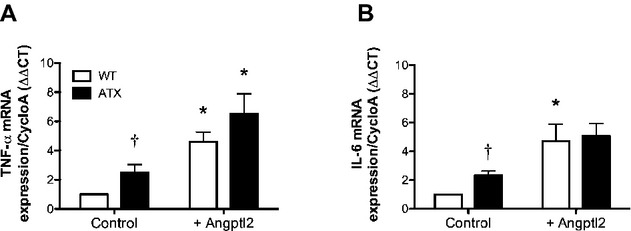
Angptl2 stimulates the expression of inflammatory markers in ECs. Quantitative RT‐PCR analysis of expression of TNF‐α mRNA (a) and IL‐6 mRNA (b) was performed in ECs freshly extracted from aortas of 3‐month‐old WT and LDLr^−/−^; hApoB^+/+^(ATX) mice stimulated and unstimulated (control) with purified recombinant angptl2 (100 nmol/L). Results were normalized to cyclophilin A (CycloA) expression, and average gene expression level in WT control cells was arbitrarily set at 1. Data are mean±SEM of n=4 WT and n=4 ATX mice; each experiment was performed in triplicate. *P<0.05 vs control; †*P*<0.05 vs WT mice. Angptl2 indicates angiopoietin like‐2; EC, endothelial cell; RT‐PCR, real‐time polymerase chain reaction; TNF‐α, tumor necrosis factor α; IL, interleukin; WT, wild type; LDL, low‐density lipoprotein; ΔΔCT, target gene threshold cycle quantification.

**Figure 2.**
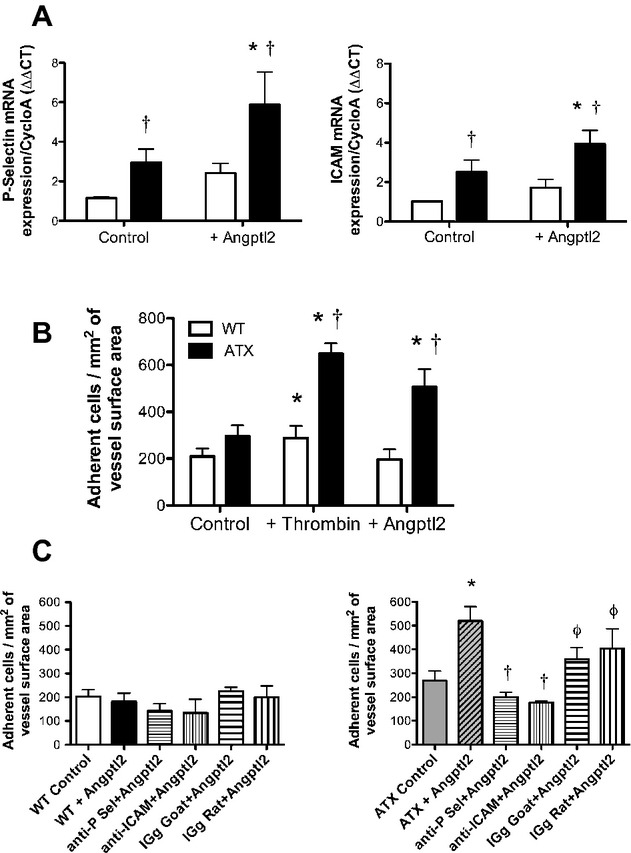
Angptl2 stimulates leukocyte adhesion to the native endothelium in ATX but not WT mice. A, Expression of P‐selectin and ICAM‐1 mRNA in freshly isolated aortic ECs from WT (n=7) and LDLr^−/−^; hApoB^+/+^ (ATX; n=6) mice was quantified by quantitative RT‐PCR and normalized to cyclophilin A after stimulation or not with purified recombinant angptl2 (100 nmol/L). Data are mean±SEM of n mice. *P<0.05 vs control; †*P*<0.05 vs condition‐matched in WT mice. B, Leukocytes from n=12 WT and n=6 ATX mice were labeled with ^51^Cr and incubated with aortic segments stimulated or not (control) with thrombin (10 U/mL) or purified recombinant angptl2 (100 nmol/L). Adherent ^51^Cr‐leukocytes were counted, and results are expressed as number of adherent cells/mm^2^ of endothelium surface area. Data are mean±SEM of n mice. *P<0.05 vs control; †*P*<0.05 vs condition‐matched in WT mice. C, Aortic segments from n=16 WT mice (left) and n=9 ATX mice (right) were preincubated with anti‐P‐selectin or anti‐ICAM‐1 antibodies or the corresponding isotype‐matched IgG prior to stimulation with purified recombinant angptl2 (100 nmol/L). Adhesion of ^51^Cr‐leukocytes was then quantified. Data are mean±SEM of n mice. *P<0.05 vs control; †*P*<0.05 vs +angptl2 condition; φ*P*<0.05 vs antibody+angptl2 condition. Angptl2 indicates angiopoietin like‐2; WT, wild type; EC, endothelial cell; LDL, low‐density lipoprotein; RT‐PCR, real‐time polymerase chain reaction; IgG, immunoglobulin G; ICAM‐1, intercellular adhesion molecule‐1.

Data are presented as mean±SEM, with “n” indicating the number of animals (Figures 1 through 7) or as dot plot (individual data for human samples, Figure 8). Gaussian distribution of the data was tested using the d'Agostino and Pearson omnibus normality test. Because of the low “n”, normality was not assessed using raw data, but using the *z* score (Y‐mean/standard deviation) of each data (Y). All data, except those in Figure 4A (data for IL‐6 expression only) passed the normality test. Therefore, except in Figure 4A where the Mann–Whitney test was used to compare IL‐6 expression in WT versus ATX mice, data were analyzed using parametric tests: the unpaired *t* test was used to compare variables in WT versus ATX mice (Figure 4) and in healthy volunteers versus CAD patients (Figure 8); 1‐way ANOVA with Tukey's post test was used to compare parameters in hIMAECs versus HUVECs and VSMCs (Figure 7), in ATX mice treated with different antibodies (Figure 2C), and in ATX mice at different ages (Figure 5A); 2‐way ANOVA with Bonferroni posttests was used to compare variables in WT versus ATX mice treated or not treated with angptl2 (Figures 1 through 3). ANOVA without repeated measures was used to compare variables in WT versus ATX mice, and 2‐way ANOVA with repeated measures was used to compare control versus +angptl2. Finally, the correlation presented in Figure 5D was tested with the Pearson test. All statistics were performed using Graph Pad Prism 5.0. A *P*<0.05 was considered statistically significant.

## Experimental Results

### Acute Stimulation With Exogenous Angptl2 Promotes Inflammation in ECs

The proinflammatory effect of angptl2 was evaluated *ex vivo* on EC freshly isolated from aortas of 3‐month‐old WT and ATX mice. Baseline mRNA levels of TNF‐α and IL‐6 were significantly higher in ECs from ATX mice (Figure 1). Stimulation of ECs with recombinant angptl2 (100 nmol/L, 30 minutes) increased (*P*<0.05) TNF‐α mRNA in both WT and ATX mice and significantly increased IL‐6 gene expression in WT mice only (Figure 1). In cultured human ECs, angptl2 (100 nmol/L) increased TNF‐α and IL‐6 gene expression after 10 minutes, an effect that was sustained after incubation of human ECs with angptl2 for 1, 4, or 24 hours (Figure S2).

### Acute Stimulation With Exogenous Angptl2 Increases P‐Selectin and ICAM‐1 Expression in EC

Inflammation is associated with upregulation of endothelial adhesion molecules. Basal expression of ICAM‐1 and P‐selectin mRNA was higher in aortic ECs freshly isolated from ATX mice than from WT mice (Figure 2A). Angptl2 stimulated the expression of ICAM‐1 and P‐selectin in ATX mice and tended to increase their expression in WT mice (Figure 2A).

### Acute Stimulation With Exogenous Angptl2 Induces Adhesion of Leukocytes Onto Native Endothelium in ATX Mice, But Not WT Mice

Expression of adhesion molecule promotes leukocyte adhesion. The basal adhesion of ^51^Cr‐leukocytes to native aortic endothelium was similar in 3‐month‐old WT and ATX mice (Figure 2B). Preincubation of the aorta endothelium with 10 U/mL of thrombin stimulated leukocyte adhesion in WT mice, and this response was potentiated in ATX mice (Figure 2B). In contrast, stimulation with recombinant angptl2 (100 nmol/L) did not induce leukocyte/endothelium interactions in WT mice, whereas it stimulated the adhesion of leukocytes in ATX mice as efficiently as did thrombin (Figure 2B). Preincubation of the aortic segments with anti‐P‐selectin or anti‐ICAM‐1 antibodies prior to stimulation with recombinant angptl2 prevented the adhesion of leukocytes on the endothelium in ATX mice (Figure 2C). Incubation with the isotype‐matched IgG did not affect angptl2‐mediated leukocyte adhesion (Figure 2C). In control experiments, aortas were incubated for 30 minutes with exogenous recombinant angptl2‐GST and then probed with an anti‐GST antibody. Subsequent analysis of immunofluorescence revealed that exogenous angptl2 efficiently bound to the aortas (Figures S3 and S4).

### Acute Stimulation With Exogenous Angptl2 Increases Adhesion Molecule Expression at Surface of the Leukocytes

**Figure 3.**
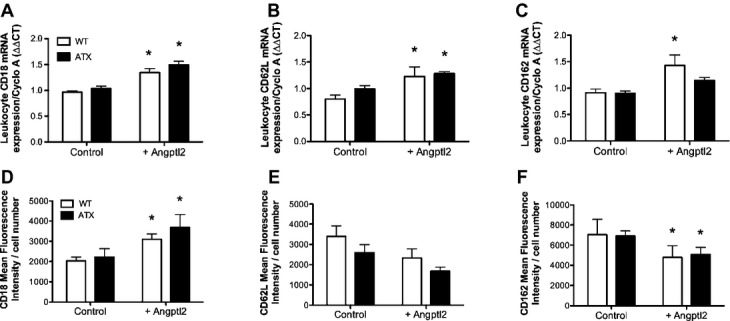
Angplt2 mRNA abundance and cell surface expression of cell adhesion molecules in leukocytes from both WT and ATX mice. Basal and angptl2‐induced mRNA expression of (A) CD18, (B) CD62L, and (C) CD162 in leukocytes from 3‐month‐old WT (n=6) and ATX (n=6) mice, were quantified by quantitative RT‐PCR and normalized by cyclophilin A. Cell surface protein expression of (D) CD18, (E) CD62L, and (F) CD162 was quantified in control and angptl2‐treated WT (n=6) and ATX (n=6) leukocytes by flow cytometry. Leukocytes were labeled using monoclonal anti‐CD18, anti‐CD62, and anti‐CD162L antibodies or with corresponding isotype‐matched IgG (data not shown). Data are mean±SEM of n mice. *P<0.05 vs control condition. Angptl2 indicates angiopoietin like‐2; WT, wild type; RT‐PCR, real‐time polymerase chain reaction.

Basal expression of CD18, CD62L, and CD162 mRNA was similar in leukocytes from WT and ATX mice (Figure 3A through 3C). Incubation with angptl2 increased the expression of these genes similarly in leukocytes isolated from WT and ATX mice (Figure 3A through 3C). Flow cytometry revealed that expression of adhesion molecule proteins was similar in leukocytes from both groups of mice (Figure 3D through 3F). Angptl2 enhanced the fluorescence intensity of CD18 similarly in WT and ATX mice (Figure 3D). Because of proteolysis of cell surface molecules, CD162 levels decreased after stimulation with angptl2 similarly in both groups (Figure 3F). Angptl2 only tended to induce CD62L shedding (Figure 3E). Angptl2 mRNA and protein were not detected in leukocytes isolated from 3‐month‐old WT or ATX mice (data not shown).

### Chronic Angptl2 Infusion Promotes Expression of Inflammatory Cytokines and Adhesion Molecules in ECs

**Figure 4.**
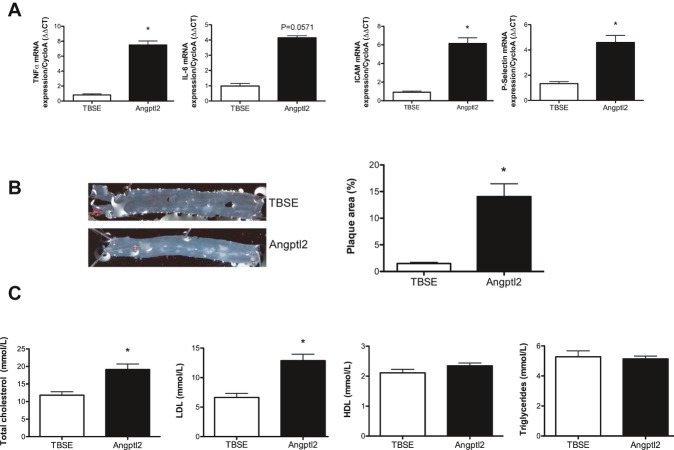
Chronic angptl2 infusion for 1 month ATX mice accelerates atherogenesis in 3‐month‐old ATX mice. A, Infusion with angptl2 promotes the expression of inflammatory cytokines and adhesion molecules in freshly scraped ECs from the aorta evaluated by quantitative RT‐PCR and normalized by cyclophilin A. B, angptl2 accelerates the formation of atherosclerotic plaque. C, angptl2 increases total cholesterol and LDL plasma levels. Data are mean±SEM of n=5 angptl2‐infused mice and n=5 TBSE‐infused mice. *P<0.05 vs TBSE condition. Angptl2 indicates angiopoietin like‐2; EC, endothelial cell; WT, wild type; RT‐PCR, real‐time polymerase chain reaction; LDL, low‐density lipoprotein; TBSE, Tris‐base, Sodium chloride, EDTA.

In preatherosclerotic dyslipidemic ATX mice, a 1‐month exposure to recombinant angptl2 (plasma concentration ≈150 ng/mL) increased the expression of inflammatory TNF‐α and IL‐6 mRNA in fresh aortic ECs by 9‐fold (*P*<0.05) and 4‐fold (*P*=0.0571), respectively (Figure 4A). In addition, the expression of ICAM‐1 and P‐selectin mRNA was increased by 7‐ and 4‐fold (*P*<0.05), respectively (Figure 4A).

### Chronic Angptl2 Infusion Accelerates Formation of Atherosclerotic Plaque

Chronic exposure of preatherosclerotic dyslipidemic mice to recombinant angptl2 led to a 10‐fold increase (*P*<0.05) in the size of the atherosclerotic lesion when compared with ATX mice infused with vehicle (TBSE; Figure 4B). Although nonsignificant lesions were observed in 4‐month‐old vehicle‐treated ATX mice (1.5±0.2% of total thoracic aorta area), angptl2 infusion increased the area of the lesions (14±2%).

### Chronic Angptl2 Infusion Increases Total Cholesterol and LDL‐Cholesterol

Chronic delivery of angptl2 significantly increased plasma levels of total cholesterol and LDL‐cholesterol without affecting HDL‐cholesterol or triglycerides (Figure 4C). Angptl2 infusion increased total cholesterol (from 11.8±1.0 to 19.1±1.6 mmol/L) and LDL‐cholesterol (from 6.6±0.7 to 12.9±1.1 mmol/L) to values similar to those in 12‐month‐old ATX mice (23.3±2.6 and 14.7±0.5 mmol/L, respectively).

### Plasma Angptl2 Levels Increase With Age and Progression of Atherosclerosis

**Figure 5.**
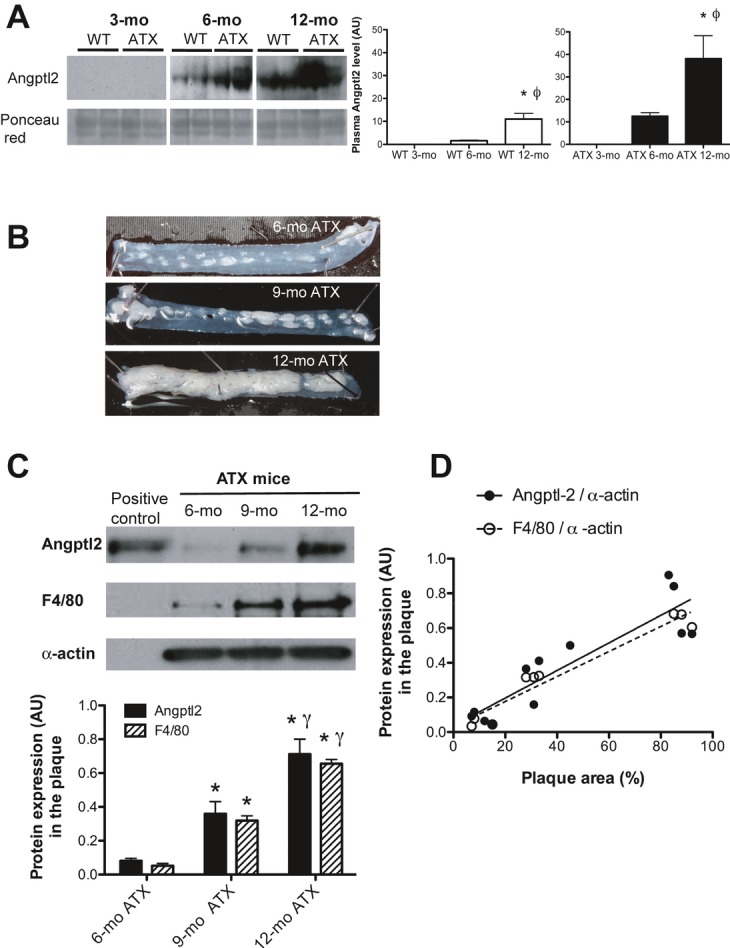
Angptl2 plasma levels and angptl2 expression in atherosclerotic plaque increase with age and atherosclerosis. A, Samples corresponding to equal amounts of total protein were collected from plasma in wild‐type (WT; n=4) and LDLr^−/−^; hApoB^+/+^ (ATX; n=4) mice at 3, 6, and 12 months of age. Low abundant proteins in the plasma samples were enriched using Bio‐Rad ProteoMiner protein enrichment kits. Following enrichment, the samples were resolved on SDS‐PAGE, transferred to nitrocellulose membranes, and probed with an antibody against angptl2. Uniform protein loading and transfer were verified by staining membranes with Ponceau red. Results (arbitrary units, AU) are presented as the mean±SEM of 4 mice in each group. *P<0.05 vs 3‐month‐old mice; ϕ*P*<0.05 vs 6‐month‐old mice. B, Photographs of atherosclerotic lesions in abdominal aortas from 6‐, 9‐, and 12‐month‐old LDLr^−/−^; hApoB^+/+^ (ATX) mice. C, Proteins were specifically extracted from the lesion, and Western blot analysis was performed for angptl2 (n=4 of 6‐, 9‐, and 12‐month‐old ATX mice) and F4/80 (n=3 of 6‐, 9‐, and 12‐month‐old ATX mice), a marker of mature macrophages. D, Correlation between angptl2 level (Pearson *r*=0.8959, *P*<0.0001, *r*^2^=0.8026, n=12), F4/80 (Pearson *r*=0.9682, *P*<0.0001, *r*^2^=0.9373, n=9), and the surface area of the lesion. Data are mean±SEM of n mice. *P<0.05 vs 6‐month‐old ATX mice; γ*P*<0.05 vs 9‐month‐old ATX mice. Angptl2 indicates angiopoietin like‐2; LDL, low‐density lipoprotein; SDS‐PAGE, sodium dodecyl sulfate polyacrylamide gel electrophoresis.

Although below the limits of detection by Western blot in 3‐month‐old WT and ATX mice, circulating levels of angptl2 increased significantly from 6 to 12 months in WT mice and even further in ATX mice (Figure 5A). In parallel, although absent at 3 months, the atherosclerotic lesion covered 10±1% of the thoracic aorta at 6 months in untreated ATX mice, then rose with age at 9 and 12 months, covering 33±4% and 74±4%, respectively, of the internal surface (Figure 5B). WT mice never developed lesions during this period.

### Endogenous Aortic Angptl2 Expression in Plaque Increases With Age and Progression of Atherosclerosis

**Figure 6.**
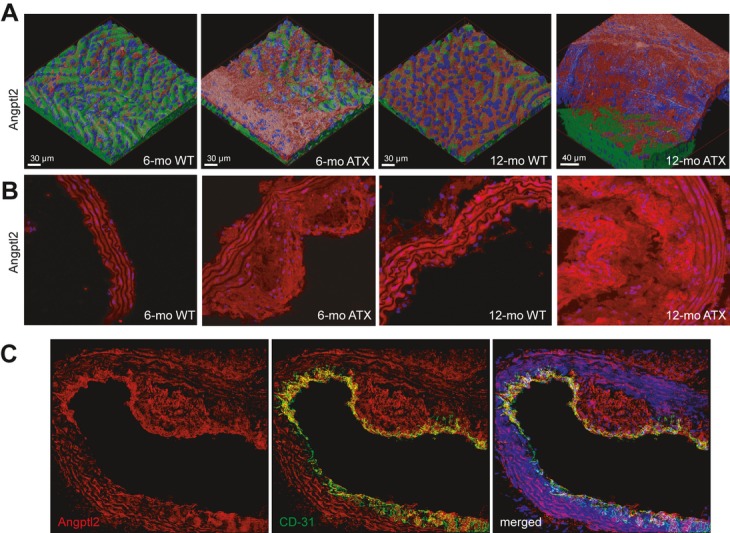
Aortic angptl2 immunofluorescence increases with age and the progression of atherosclerosis, with marked accumulation within atherosclerotic plaque. Immunofluorescence was used to visualize angptl2 in (A) fresh longitudinally opened aortas and (B) frozen aortic sections of WT and LDLr^−/−^; hApoB^+/+^ (ATX) mice at 6 and 12 months of age. A, z‐stacks were acquired, deconvolved, and 3D images rendered. Angptl2 accumulates in plaque and in media. Angptl2 levels are shown in red and basal membrane in green. Nuclei are shown in blue. C, Double‐immunostaining of angptl2 (red) and CD‐31 (green) in frozen aortic sections from 6‐month‐old ATX mice, showing that angptl2 colocalizes with the endothelial marker CD‐31. Angptl2 indicates angiopoietin like‐2; WT, wild type; LDL, low‐density lipoprotein.

In ATX mice, angplt2 protein expression was quantified specifically in the aortic lesion: its pattern of expression positively correlated with the lesion area and followed the expression of F4/80, a specific marker of macrophages (Figure 5C and 5D). WT mice did not develop lesions (Figure 6), but endogenous angptl2 aortic mRNA and protein expression increased with age from 6 to 12 months in WT mice, although to a lesser extent than in ATX mice (data not shown).

Confocal immunofluorescent images showed that the endogenous levels of angptl2 increased with age and the progression of atherosclerosis (Figure 6A and 6B). This was observed in both freshly isolated, longitudinal aortic sections (Figures 6A and S5) and in frozen sections (Figures 6B and S5). Angptl2 was particularly abundant in the atherosclerotic lesion but also present in ECs, as demonstrated by the colocalization of angptl2 with the endothelial marker CD‐31 (Figures 6C and S3). Angptl2 fluorescence in VSMCs was also clearly observed throughout the media in 6‐ and 12‐month‐old WT and ATX mice (Figures 6B and 6C and S3 and S4), but not in young 3‐month‐old WT mice and at a very low level in preatherosclerotic 3‐month‐old ATX mice (Figure S6).

### Angptl2 Is Secreted by ECs But Not by VSMCs

**Figure 7.**
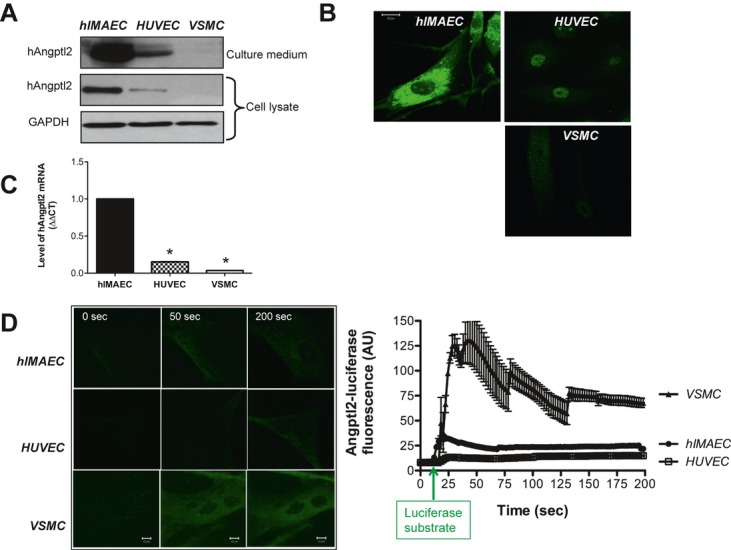
Expression and secretion of angptl2 is greater in ECs than in VSMCs, but angptl2 binding is higher in VSMCs than in ECs. A, Western blot analysis of endogenous angptl2 secreted into the culture medium overnight (16 h) by hIMAECs, HUVECs, and VSMCs. Angptl2 protein expression was also quantified in the cell lysate. B, Endogenous angptl2 expression in the cells was detected by immunofluorescence using a confocal microscope. Scale bar represents 20 μm. C, Quantitative RT‐PCR analysis of angptl2 mRNA expression was performed in the cell lysate from cultured cells (n=3 hIMAECs, n=3 HUVECs, n=3 VSMCs). Results were normalized to GAPDH expression, and average gene expression in hIMAECs was arbitrarily set at 1. Data are mean±SEM of n=3; each experiment was performed in duplicate. *P<0.05 vs hIMAECs. D, To assess angptl2 binding, hIMAECs, HUVECs, and VSMCs were incubated for 10 minutes with human angptl2‐luciferase (100 nmol/L) in phenol red‐free medium. Cells were then washed, and the binding of angptl2‐luciferase to cell surface was revealed by adding the luciferase substrate. A selection of confocal time‐series images acquired in a single living cell at 0, 50, and 200 seconds is shown (left). The average angptl2‐luciferase fluorescent signals (AU) recorded in VSCMs (n=6 cells), hIMAECs (n=6 cells), and HUVECs (n=4 cells) were derived from the time‐series images (right). Data are mean±SEM, and the assay was performed 3 to 4 times. EC indicates endothelial cell; VSMC, vascular smooth muscle cell; hangptl2, human angiopoietin like‐2; hIMAEC, human internal mammary artery endothelial cell; HUVEC, human umbilical vein endothelial cell; RT‐PCR, real‐time polymerase chain reaction; AU, arbitrary unit.

To elucidate the cellular specificity of angptl2 expression, we compared its production by cultured human ECs isolated in coronary patients (hIMAECs),^23^ HUVECs, and human VSMCs. Immunoblotting analysis (Figure 7A) showed that angptl2 protein was abundantly expressed and secreted by hIMAECs, less detectable in HUVECs, and undetectable in VSMCs. Similar results were observed by immunofluorescence (Figure 7B) and at the mRNA level (Figure 7C). These results indicate that angptl2 is secreted by ECs, particularly in a proatherosclerotic environment, but not by VSMCs. It is likely that angptl2 expression is higher in hIMAECs than in HUVECs because of the elevated level of damage sustained by cells isolated from patients with severe atherosclerosis.^23^

### Intensity of Angptl2‐Luciferase Binding Is Stronger in VSMCs Than in ECs

The binding of recombinant angptl2‐luciferase to the cell surface of living hIMAECs, HUVECs, and VSMCs was examined (Figure 7D). The binding of luciferase alone (no angptl2 in the construct) was negligible (Figure S7). We observed a marked accumulation of angptl2‐luciferase on the surface of living VSMCs over time; the signal remained stable for up to 5 minutes. The signal was less intense on the surface of HUVECs and hIMAECs (Figure 7D). These results suggest that angptl2‐luciferase is more efficiently captured by VSMCs than by ECs, implying that angptl2 receptors are in greater abundance and/or affinity on VSMCs than on ECs. Altogether, the data from Figure 7 show that angptl2 is produced by ECs, but not by VSMCs, and that it predominantly binds to VSMCs (Figures S3 and S4).

### Circulating Levels of Angptl2 Increase in CAD Patients

**Figure 8.**
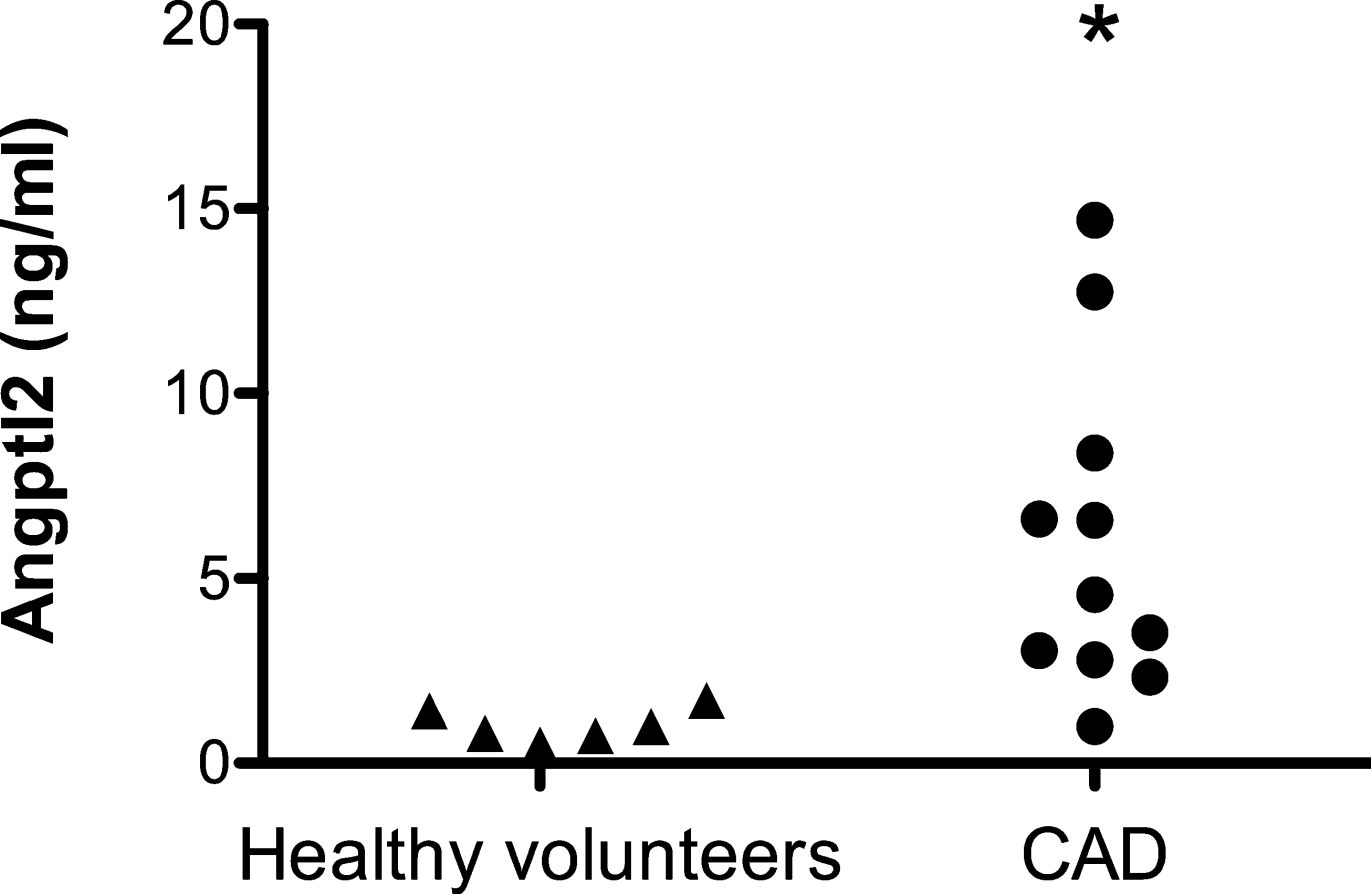
Plasma angptl2 levels are higher in CAD patients than in healthy volunteers. Circulating angptl2 levels were quantified in CAD patients (n=11) and in age‐matched healthy volunteers (n=6) by ELISA. Data are presented as a dot plot. *P<0.05 vs healthy volunteers. Angptl2 indicates angiopoietin like‐2; CAD, coronary artery disease; ELISA, enzyme‐linked immunosorbent assay.

Compared with age‐matched male healthy volunteers (Table), plasma angptl2 levels were significantly higher in CAD patients (1.00±0.18 versus 6.02±1.33 ng/mL, healthy subjects versus CAD patients; Figure 8). The presence of CAD was documented by a history of angina (5 of 11 patients), infarct (6 of 11), previous dilatation (3 of 11), or coronary bypass (1 of 11). Almost all CAD patients were dyslipidemic and treated with statins (10 of 11), explaining their lower total cholesterol and LDL‐cholesterol levels when compared with healthy volunteers (Table).

## Discussion

The novel findings of this study are that (1) angptl2 promotes the adhesion of leukocytes to the native inflamed endothelium of preatherosclerotic young ATX mice, but not WT mice, via a robust activation of P‐selectin and ICAM‐1; (2) the chronic administration of angptl2 in preatherosclerotic young ATX mice strongly accelerates the formation of atherosclerotic lesions while increasing the expression of endothelial proinflammatory cytokines and adhesion molecules and circulating levels of cholesterol; (3) angptl2 plasma levels increase with age and disease, angptl2 concentrates in the atherosclerotic lesion of aging ATX mice and binds predominantly to VSMC; and (4) angptl2 is secreted by cultured ECs but not by VSMCs. We have also shown for the first time that circulating levels of angptl2 are significantly higher in white CAD patients than in healthy volunteers. Altogether, these observations demonstrate that angptl2 is active in the different steps of atherogenesis, promoting leukocyte adhesion to the inflamed preatherosclerotic and dysfunctional^22^ vascular endothelium and contributing to the formation of the lesions.

Studies investigating the physiological and pathophysiological role of angptl2 are limited. It has been reported that angptl2 is a growth factor that stimulates the expansion^10^ and survival^24^ of hematopoietic stem cells. It has been shown to be proangiogenic^2,12^ and to induce vasculogenesis in mice,^8^ and to be proinflammatory.^11,15–19,25^ Despite strong evidence that angptl2 is positively associated with chronic inflammatory diseases, its direct implication in atherogenesis is unknown. Circulating angptl2 levels were higher in Japanese patients with CAD when compared with healthy subjects and correlated with severity of the disease.^8,11^ In the present study, we have shown that white CAD patients also exhibit higher angptl2 plasma concentrations than healthy volunteers (Figure 8). Interestingly, although the values of angptl2 levels were similar in healthy caucasian and Japanese subjects, circulating levels of angptl2 were higher in our North American cohort of CAD patients than in Asian CAD patients.^8,11^

To study the role of angptl2 in atherogenesis, we used LDLr^−/−^; hApoB_100_^+/+^ mice characterized by severe dyslipidemia, that is, abnormally high circulating levels of total cholesterol (8‐fold), LDL‐cholesterol (20‐fold), and triglycerides (9‐fold).^21–22,26^ Three‐month‐old ATX mice exhibit premature endothelial dysfunction, oxidative stress, inflammation; ATX mice develop aortic atherosclerotic plaques by the age of 6 months^21–22,26^ (Figures 5 and 6). At 3 months, ATX mice are therefore prone to react to a proatherosclerotic factor. Consistent with the established relationship between inflammation and the progression of atherosclerosis,^27–28^ we observed that recombinant angptl2 upregulated the inflammatory cytokines TNF‐α and IL‐6 in ECs freshly isolated from the aortas of WT and ATX mice and within 10 minutes in cultured human ECs (Figure S2). Similarly, a recent study^11^ focusing on the proinflammatory role of angptl2 showed that plasma angptl2 levels correlated with inflammation, adiposity, and insulin resistance both in mice and humans. Although basal levels of TNF‐α and IL‐6 were higher in the endothelium of ATX compared with WT mice, we showed that the upregulation of both inflammatory cytokines by acute exposure to angptl2 was not exacerbated in ATX mice (Figure 1). However, chronic infusion of angptl2 strongly potentiated the inflammatory response of the endothelium in preatherosclerotic mice (Figure 4). This potent proinflammatory effect of angptl2 likely synergizes with the rise in total cholesterol to accelerate atherogenesis.

Atherogenesis involves 3 essential successive phases, starting with (1) capture and rolling of leukocytes from circulation to the inflamed endothelium, (2) arrest and firm adhesion of leukocytes, and (3) transendothelial migration of leukocytes.^29^ Our data demonstrate for the first time that angplt2 promotes leukocyte adhesion to the native endothelium *ex vivo* and that this property of angptl2 requires a proinflammatory environment. Indeed, angptl2 stimulated leukocyte adhesion in ATX mice, but not in WT mice (Figure 2). The proinflammatory environment of the ATX mice (illustrated by increased expression of inflammatory and adhesion molecules, Figures 1 and 2A) likely primes ECs to strongly respond to angptl2. Importantly, leukocyte adhesion induced by angptl2 was prevented by P‐selectin or ICAM neutralization. P‐selectin deficiency has been associated with a reduction in inflammation and in the progression of atherosclerosis in ApoE‐null mice.^30^ In addition, P‐selectin inactivation limits plaque macrophage content and neointima formation after endothelial injury in the carotids of ApoE‐null mice.^31^ Altogether, these data further support the essential role of inflammation, leukocyte adhesion, and macrophages in the initiation of the atherosclerotic process in dyslipidemic mice. In contrast, angptl2 increased the expression of adhesion molecules at the surface of leukocytes isolated from WT and ATX mice to a similar extent (Figure 3), suggesting that the increase in cell adhesion observed in the native endothelium from ATX mice was not a result of altered adhesion molecule expression on leukocytes, but rather of upregulation of adhesion molecule expression on ECs. Altogether, our data^14^ and those from the literature^15–16,19^ suggest that chronic inflammation may trigger angptl2 expression and feed‐forward an endothelial proinflammatory loop^30,32^ and initiate atherogenesis through P‐selectin‐ and ICAM‐dependent leukocyte adhesion.

In a seminal study, it was briefly reported that in mice overexpressing angptl2, a model associated with inflammation, adhesion of leukocytes/macrophages to the vascular wall was augmented.^11^ The authors proposed that the fibrinogen‐like domain of angptl2 might interact with the integrin α5β1 expressed at the site of inflammation.^11^ In our ATX mice, however, we could not detect integrin α5β1 mRNA expression in native aortic ECs whether exposed or not to angptl2 (data not shown). Angptl2 is still considered an orphan ligand, although a recent study demonstrates that immune inhibitory receptors such as human leukocyte immunoglobulin‐like receptor B2 bind different angptls including angptl2.^33^

Chronic exposure (1 month) of young preatherosclerotic ATX mice to angptl2 induced a potent inflammatory response, potentiated the expression of endothelial adhesion molecules, and strongly accelerated the formation of atherosclerotic lesions (Figure 4). Unexpectedly, angptl2 also significantly increased both total cholesterol and LDL‐cholesterol levels. Although angptl3,^4–5^ angptl4,^6^ and angptl8^3^ are known to modulate lipid metabolism, to the best of our knowledge, this is the first report of such an effect of angptl2 on circulating cholesterol levels. A recent study, however, demonstrated that levels of angptl2 reflect adiposity and might regulate inflammation and triglyceride metabolism.^34^ Although inflammation triggers atherosclerosis in animal models of dyslipidemia,^32^ it is not possible at this stage to decipher whether the premature atherogenic response induced by angptl2 is a result of its proinflammatory or its hypercholesterolemic effect. We observed that the infusion of angptl2 in ATX mice increased total cholesterol levels by 62% (Figure 4C); whether this augmentation alone, on top of an already very high level of cholesterol (7‐fold increase), could accelerate atherosclerotic plaque formation by ≈10‐fold (Figure 4B) is possible, but unlikely. However, the combined hypercholesterolemic and proinflammatory effects of angptl2 (Figure 4A) likely synergize to promote atherosclerosis. The relative contribution of inflammation versus hypercholesterolemia in atherogenesis is a recurrent question in the field of atherosclerosis.^35–36^ Short‐term intervention with sulfasalazine, an inhibitor of NFκB, in patients with coronary artery disease has not been successful in reversing endothelial dysfunction,^37^ whereas in a small group of patients with recent acute coronary syndrome, inhibition of the leukotriene‐generating enzyme 5‐lipoxygenase reduced inflammation and plaque progression.^38^ More studies are ongoing to further decipher the exact contribution of inflammation in atherogenesis.^28,36^

Both circulating and aortic levels of angptl2 increased progressively with aging and prematurely with atherosclerosis. Consistently, healthy aging is associated with a rise in blood levels of angptl2 in humans,^11^ whereas in diabetic and obese subjects, a positive correlation was found between the circulating level of angptl2 and the concentration of the biomarker C‐reactive protein.^11^ In addition, we observed a strong increase in endogenous angptl2 expression in human ECs isolated from patients undergoing coronary artery bypass surgery (Figure 7A through 7C) characterized by premature senescence and a highly proatherogenic environment^14,23^; after chronic treatment of these cells with the antioxidant N‐acetyl‐cystein, the expression of angptl2 was reduced (unpublished data). Interestingly, angptl2 expression is higher in hIMAECs than in HUVECs (Figure 7), likely because of the elevated level of oxidative damage sustained in vivo.^23,39^ The elevation in circulating angptl2 associated with the progression of atherosclerosis in ATX mice (Figure 5A) and its accumulation in plaque (Figures 5B and 5C and 6A and 6B) strongly suggest that angptl2 contributes to the pathology. Although we have shown that cultured VSMCs did not secrete angptl2, confocal immunofluorescent images showed high levels of angptl2 not only in the plaque but also in the endothelium and throughout the media. Angptl2 production could differ between cultured cells and in vivo; however, this is unlikely because cultured VSMCs have a secretory phenotype. On the other hand, our data suggest that the angptl2 present in VSMCs is a result of its binding, but not its local production. Indeed, immunohistofluorescent staining in aortic sections of 3‐month‐old WT mice showed that angptl2 was absent in VSMCs and mostly present in ECs (Figure S6). In ATX mice of the same age, however, angptl2 was observed in VSMCs in addition to in ECs. Circulating angptl2 may originate from various cell types, including EC, adipocytes, and macrophages, and bind to ECs, VSMCs, and other cells in the plaque. In line with this, plasma angptl2 levels increase with age and even more in ATX mice (Figure 5A). Thus, it is possible that circulating angptl2 binds to VSMCs to a greater extent in ATX mice (Figure 6). The biological consequences of the binding on VSMCs remain to be elucidated.

Angptl2 can be produced by thioglycollate‐induced peritoneal macrophages, inducing vascular inflammation and contributing to the formation of abdominal aneurysms.^19^ We observed a parallel rise in protein expression of angptl2 and of macrophage F4/80 in the plaque (Figure 5B through 5D), but we do not know if macrophages from the plaque produce angptl2. We observed that leukocytes do not express angptl2 (data not shown), but according to the literature,^19^ their transformation to macrophages should be accompanied by the induction of angptl2 expression.

Further studies are needed to decipher whether T cells or any cell type contained in plaque other than ECs and macrophages could contribute to angptl2 expression within atherosclerotic lesions. Similarly, the specific cell type within leukocytes that binds the endothelium and infiltrates the wall should be identified; indeed, leukocytes include many different cell types such as monocytes, effector and regulatory T cells, and neutrophils; thus, using leukocytes instead of pure monocytes could be a limitation. The present study focused on TNF‐α and IL‐6 as inflammatory markers and P‐selectin and ICAM‐1 as adhesion markers, although a broader spectrum of inflammatory cytokines, growth factors, and adipokines could be involved in the observed proatherogenic effect of angptl2. That some comparisons failed to reach statistical significance, because of small sample sizes, is another potential limitation. Finally, other models of atherosclerosis must be examined to validate that angptl2 also contributes to the pathogenesis of atherosclerosis. Nonetheless, as angptl2 levels are significantly higher in white (Figure 8) and Japanese CAD patients,^11^ and based on our present data, we believe that our findings are not limited to ATX mice.

In conclusion, our results demonstrate that angptl2 increases in the blood with age in mice and prematurely in ATX mice. We found that angptl2 is a key inflammatory factor activating adhesion molecule expression while increasing circulating cholesterol levels, accelerating the development of atherosclerotic plaques in preatherosclerotic, severely dyslipidemic mice. Although angptl2 is still an orphan ligand, more studies are needed to decipher the exact mechanisms of action of angptl2, which may emerge as a key player in the atherosclerotic process.
